# Prevalence of *Trypanosoma congolense* and *Trypanosoma vivax* in Lira District, Uganda

**DOI:** 10.1155/2021/7284042

**Published:** 2021-06-14

**Authors:** Aziz Katabazi, Adamu Almustapha Aliero, Sarah Gift Witto, Martin Odoki, Simon Peter Musinguzi

**Affiliations:** ^1^Department of Microbiology and Immunology, Faculty of Biomedical Sciences, Kampala International University-Western Campus, P.O. Box 71 Bushenyi, Uganda; ^2^Department of Microbiology and Immunology, Division of Biomedical Sciences, Kabale University School of Medicine, P.O. Box 315 Kabale, Uganda; ^3^Department of Animal Sciences, Faculty of Agriculture and Environmental Sciences, Kabale University, P.O. Box 317 Kabale, Uganda

## Abstract

Trypanosomes are the causative agents of animal African trypanosomiasis (AAT) and human African trypanosomiasis (HAT), the former affecting domestic animals prevalent in Sub-Saharan Africa. The main species causing AAT in cattle are *T. congolense*, *T. vivax*, and *T. b. brucei*. Northern Uganda has been politically unstable with no form of vector control in place. The return of displaced inhabitants led to the restocking of cattle from AAT endemic areas. It was thus important to estimate the burden of trypanosomiasis in the region. This study was designed to compare the prevalence of animal African trypanosomes in cattle in Lira District using microscopy and polymerase chain reaction amplification (PCR) methods. In this cross-sectional study, a total of 254 cattle from the three villages of Acanakwo A, Barropok, and Acungkena in Lira District, Uganda, were selected by simple random sampling technique and screened for trypanosomiasis using microscopy and PCR methods. The prevalence of trypanosomiasis according to microscopic results was 5/254 (2.0%) as compared to 11/254 (4.3%) trypanosomiasis prevalence according to PCR analysis. The prevalence of trypanosomiasis infection in the animal studied is 11/254 (4.3%). *Trypanosoma congolense* was the most dominant trypanosome species with a proportion of 9/11 (81.8%), followed by *T. vivax* 1/11 (9.1%) and mixed infection of *T. congolense*/*T. vivax*1/11 (9.1%). Barropok village had the highest prevalence of trypanosomiasis with 6/11 (54.5%). There is a statistically significant relationship (OR = 6.041; 95% CI: 1.634-22.331; *p* < 0.05) between abnormal PCV and trypanosome infection. Polymerase reaction amplification was the most reliable diagnostic method due to its high sensitivity and specificity as compared to the conventional microscopic method. Polymerase reaction amplification appears to have adequate accuracy to substitute the use of a microscope where facilities allow. This study, therefore, underscores the urgent need for local surveillance schemes more especially at the grassroots in Uganda to provide data for reference guideline development needed for the control of trypanosomiasis in Uganda.

## 1. Introduction

Animal African trypanosomiasis (AAT) is one of the most significant vector-borne diseases of domestic animals in the tsetse belt of Africa including East Africa [1]. AAT is caused by *Trypanosoma* species which are transmitted cyclically by the tsetse fly belonging to the *Glossina* spp. Animal African trypanosomiasis (AAT) affects a wide range of hosts, namely, goats, sheep, donkeys, and cattle. The disease is characterized by the presence of parasites in the blood and alternating fever [2]. Anaemia usually develops in infected animals, accompanied by weight loss, body loss, miscarriage, abortion, decreased productivity, and sometimes mortality [3]. Animal African trypanosomiasis causes more than 3 million animals to die each year with 50 million animals at risk of infection [4]. Therefore, AAT is the main hindrance to food security, as it makes vast areas of semiarid savannah land in Africa unsuitable for breeding domestic animals that are a source of dairy and meat products [5]. Also, AAT remains a setback in most livestock-dependent economies in Sub-Saharan Africa, causing economic losses of agricultural productivity of about 20% [6], and because most livestock rearing is commonly practised by rural communities, AAT impedes rural development [7].

In livestock, AAT is caused by *Trypanosoma congolense*, *Trypanosoma vivax*, *Trypanosoma evansi*, *Trypanosoma simiae*, and *Trypanosoma brucei brucei* [8]. *Trypanosoma* species infection in cattle is primarily spread through bites of infected tsetse flies found in over 37 African countries in Sub-Saharan Africa including Uganda [9]. Other biting flies such as *Stomoxys* have also been implicated in the spread of AAT although they lack epidemiological significance [10]. Several host factors, including the physiological status of the host and nutritional and environmental factors, have an important effect on pathogenicity and determine the severity of the disease [11]. Illness in animals affected with *T. congolense*, *T. vivax*, or *T. b. brucei* is characterized by fluctuating parasitaemia with periods of paroxysms and intermission. This leads to anaemia; the roughness of the hair coat, abortion, reduced milk yield, intermittent pyrexia, depression, and gradual loss of condition lead to extreme emaciation and death of the animal [12].

Diagnosis is an important factor in the controlling of infection. Several diagnostic techniques for trypanosomiasis exist; however, only a few tests have been objectively analyzed and standardized. Trypanosomiasis has traditionally been diagnosed using microscopy to directly observe the *Trypanosoma* parasites in blood either through a wet film system for detecting mobile trypanosomes or as thick and thin dried smears. Recently, with the explosion of new techniques, because of the rapid developments in molecular biology, new approaches and technologies can be used for diagnosis [13]. The use of molecular methods such as polymerase chain reaction (PCR) has helped to diagnose and classify *Trypanosoma* species [14].

There are several methods for detecting trypanosomiasis in animals, including parasitological, immunological, and molecular methods [15, 16]. This study used traditional microscopy and molecular techniques to quantify the prevalence and identify the circulating species.

## 2. Materials and Methods

### 2.1. Study Area

**Figure 1:**
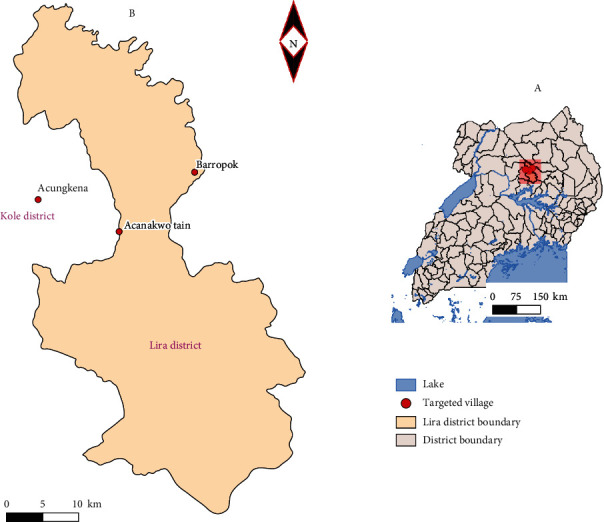
Map of Uganda showing the studied area: Lira District (Uganda Bureau of Statistics Copyright ©2018).

This study took place in Lira District, Uganda (Figure 1), which is located in the following coordinates: 2°14′50.0^″^N 32°54′00.0^″^E (latitude: 02.2472; longitude: 32.9000) in Northern Uganda [17]. Lira District is the main economic hub in northern Uganda, and livestock keeping is one of the main economic activities. Furthermore, Lira District is one of the districts that benefitted from the restocking program funded by the African Development Bank (ADB). In 2008, the national livestock census reported the cattle population to be 15,933. This put Lira District among the cattle rearing districts which rendered it being an AAT hotspot. The cattle were sampled from Acungkena, Barropok, and Acanakwo A subcounties.

### 2.2. Sample Size Determination

This study used a survey formula previously reported by Kish [18]: *n* = *z*^2^*p* (1 − *p*)/*d*^2^, where *z* is the *Z* score for 95%confidence interval = 1.96, *p* is the prevalence, and *d* is the acceptable error (5%). We used the prevalence of bovine African trypanosomes15.3% in Tororo District, southeastern Uganda [1].

### 2.3. Study Design

This was a cross-sectional study aimed at establishing the prevalence of Animal African trypanosomes in Lira District using two selected diagnostic methods. A simple random sampling technique was used to identify the study sites. Two hundred and fifty-four (254) animals were selected and assigned unique numbers for identification according to the village and farm that they came from. This study was carried out from March 2020 to April 2020.

### 2.4. Sample Collection

From each selected animal, the blood was collected from the jugular vein into vacutainer tubes containing EDTA and stored at 4°C in a cooler box and transported to the district laboratory for microscopy analysis and sample processing for molecular analysis. For molecular analysis, the blood from the vacutainer tubes was applied onto Whatman FTA cards and left to dry and then stored in sealed plastic bags at room temperature. To have higher chances of detecting parasites, blood sample collection was done in the morning [19].

### 2.5. Microscopy

For microscopy, 50 *μ*l of whole blood was used to make thin and thick blood films. The blood films were allowed to air dry and then fixed in concentrated methyl alcohol for about 1-2 minutes followed by staining with 10% Giemsa for 25-35 minutes. The blood films were then washed with distilled water to remove the excess stain, and the slides were left to air dry before viewing under an oil immersion lens [20].

### 2.6. DNA Extraction

DNA was extracted with slight modification as previously described by [9]. A 6 mm diameter disc was cut from the blood spot on the Whatman FTA card and placed into the 0.5 ml microcentrifuge tube. 200 *μ*l of sterile water was added, and the tube incubated at 37°C for 30 min in a heating block. The tubes were then centrifuged at high speed (14000 RPM) for 1 minute, and the resulting supernatant was collected and discarded.

A second 100 *μ*l aliquot of sterile water was added to the Whatman FTA card discs in the original tube and incubated at 100°C for 30 min in the heating block. After incubation, the sample was centrifuged at 14000 RPM for 1 minute. The supernatant was removed and stored at -20°C ready for PCR analysis.

### 2.7. Polymerase Chain Reaction (PCR)

PCR was performed with slight modification as previously reported by Njiru et al. [21] using a 25 *μ*l reaction volume containing 1 gm of Taq (10 mM Tris-HCl, 50 mM KCl, 1.5 mM MgCl2, 2.5 units of pure Taq DNA polymerase, 200 *μ*M of each of the four deoxynucleoside triphosphates, and reaction buffer), 1 *μ*l of each primer (1 *μ*M), 2 *μ*l DNA sample, and double distilled water to a final volume of 25 *μ*l. *Trypanosoma brucei brucei* reference DNA was used as a positive control, and nuclease-free PCR water was used as a negative template control. The cycling conditions that were used are as follows: first denaturation at 98°C for 1 min, denaturation at 98°C for 5 seconds, annealing at 64°C for 30 seconds, extension at 72°C for 30 seconds (25 cycles), and the final extension for 72°C for 10 mins [21]. The PCR conditions and cycling conditions were the same for both the 1st and 2nd PCR runs. The amplified PCR products were run on a 2% agarose gel, stained in 0.5 *μ*g/ml ethidium bromide solution, and viewed using a UV illuminator and documented. The sequences of the primers used in the amplification and expected base pairs size of each *Trypanosoma* species are shown in Table 1.

### 2.8. Data Analysis

Data were entered in Microsoft Office Excel 2007 and then exported to IBM SPSS version 20. Descriptive statistic was used to determine the prevalence and distribution of trypanosome species in the villages.

### 2.9. Ethical Consideration

The study was carried out according to the guidelines stated in the Kampala International University Research Ethics Committee Manual of 2017. Only animals whose owners consented were recruited into the study. All animals were handled under the considerations of the National Institute of Health guidelines for the care of animals with the help of a veterinary assistant.

## 3. Results

### 3.1. Prevalence of Trypanosome Infections in Cattle

A total of 254 cattle from Acungkena, Barropok, and Acanakwo A subcounties were screened. Of these, 107 cattle were positive for trypanosomes by hematocrit centrifugation technique (HCT) (Table 2). There was a variation in the sample size between the two subcounties because of the cattle populations per subcounty.

The prevalence of trypanosomiasis according to microscopic results was 5/254 (2.0%) as compared to 11/254 (4.3%) trypanosomiasis prevalence according to PCR analysis (Tables 3 and 4). Microscopy demonstrated that Barropok village had the highest prevalence of trypanosomiasis with 3/5 (60.0%) followed by village 1/5 (20.0%) and village 1/5 (20.0%) (Table 3).

When the PCV results were compared to trypanosomiasis status, *T. congolense* distribution between normal and abnormal PCV range distribution was 6/9 (66.7%) and 3/9 (33.3%), respectively (Table 2). Infection with *T. vivax* and mixed infection (*T. congolense*/*T. vivax*) showed 1/1 (100.0%) trypanosomiasis status in the normal and abnormal PCV ranges, respectively. Noninfected animals demonstrated 21/243 (8.6%) and 222/243 (91.4%) in the abnormal and normal PCV ranges, respectively (Table 2).

**Figure 2:**
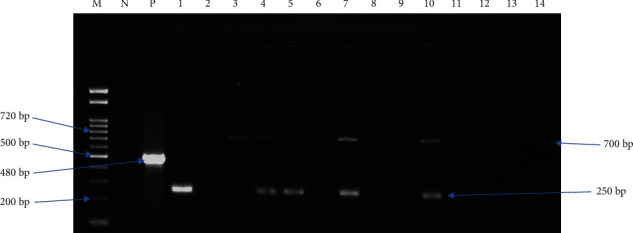
Electrophoretic analysis of ITS PCR products. Representative agarose gel results showing the different sizes of the ITS1 region for the different trypanosome species (*T. brucei* -480 bp, *T. vivax* -250 bp, and *T. congolense* -700 bp) amplified using nonnested primers. Trypanozoon that gives an expected band size of 480 bp was not detected. Lane M: 100 bp DNA marker; Lane N: negative control (sterile water with no template added); Lane P: positive control (*T. b. b* GVR-35); Lanes 1-14: test samples.

Polymerase chain reaction amplification demonstrated that the prevalence of trypanosomiasis infection in the animal studied is 11/254 (4.3%). Barropok village had the highest proportion of trypanosomiasis with 6/11 (54.5%), followed by Acanakwo A village2/11 (18.2%) and Acungkena village 3/11 (27.3%) (Table 5). *Trypanosoma congolense* was the most dominant trypanosome species with a proportion of 9/11 (81.8%), followed by *T. vivax* 1/11 (9.1%) and mixed infection of *T. congolense*/*T. vivax*1/11 (9.1%) (Table 4 and Figure 2).

There is statistical significant relationship (OR = 6.041; 95% CI: 1.634-22.331; *p* < 0.05) between abnormal PCV and trypanosomiasis infection. This result also indicates that the animal with a PCV volume ≤ 29 is at risk of trypanosomiasis infection (Table 6).

## 4. Discussion

Trypanosomiasis is one of the leading haemoparasitic diseases found in animals reared at home. Trypanosomiasis is transmitted by the tsetse fly species known as the Glossina, and it is widespread all over the tropical regions of Africa that exhibit vector prevalence. The main trypanosome species transmitted by the tsetse fly are *Trypanosoma congolense*, *T. vivax*, and *T. brucei* in sheep, goats, and cattle and *T. simiae* in pigs. However, little knowledge about the prevalence and diversity of trypanosomes circulating in upcountry districts in northern Uganda which lies in the tsetse fly belt [9] has been a problem making AAT management hard. Moreover, the effectiveness of AAT diagnostic methods used popularly by the animal health professionals in those northern districts is not known. This study, therefore, compared the presence of animal African trypanosomes in cattle in Lira District using microscopy and molecular methods.

The prevalence of trypanosomes according to microscopy results was 5/254 (2.0%) as compared to 11/254 (4.3%) trypanosomiasis prevalence according to PCR analysis. The microscopic results of this study are comparable to reports elsewhere [21, 22], and the PCR results that demonstrated a slightly higher prevalence in this study agree with previous reports [22, 23]. The higher prevalence of trypanosomiasis shown by using the PCR method is because the molecular technique is more sensitive and specific as compared to the conventional microscopic methods [24]. The substantial sensitivity of the PCR method over the conventional microscopic method in this study conforms to a previous study [22, 23]. Most studies have used microscopy as the reference standard. However, microscopy has relatively low sensitivity although most studies have used centrifugation to upsurge sensitivity [24].

*Trypanosoma congolense* was the most common *Trypanosoma* species with 9/11 (81.8%), followed by *T. vivax* 1/11 (9.1%) and mixed infection of *T. congolense*/*T. vivax*1/11 (9.1%). The high proportion of *T. congolense* 9/11 (81.8%) demonstrated in this study as compared to *T. vivax* 1/11 (9.1%) is in agreement with previous reports [1]. *Trypanosoma congolense* is known to be the leading aetiology of trypanosomiasis of the salivarian livestock group that is highly prevalent in Sub-Saharan Africa and results into a huge negative impact on the economy on the affected countries where it is endemic [25]. This parasite causes a disease called Nagana, which means “depressed spirit” according to the Zulu of South Africa.

This study also showed a significantly low prevalence of 1/11 (9.1%) of mixed infection caused by *T. congolense*/*T. vivax*. The significantly low prevalence of 1/11 (9.1%) of mixed infection prevalence demonstrated is conformity with other previous reports [22]. The presence of mixed infection in this study is supported by previous studies done elsewhere [25, 26], though the low prevalence of mixed infection demonstrated in this is not in line with other previous studies in other regions of Africa where a high prevalence of trypanosomiasis due to mixed infections has been reported [1, 23]. The variations in the prevalence of mixed infections involving different species of trypanosome from one place to another are probably due to the accessibility of trypanosome species to their specific hosts. Even though little is known about the factors determining the spread and abundance of trypanosome species, the presence of a suitable mammalian host remains the most likely factor [27].

In addition to *T. congolense* species with the highest proportion of 9/11 (81.8%) among AAT disease complex in the Lira District, the current investigation revealed 1/11 (9.1%) and 1/11 (9.1%) proportions of *T. vivax* and *T. congolense*/*T. vivax*, respectively. This research further demonstrates that T*. brucei brucei*, *T. vivax*, and *T. congolense* are still present in livestock reservoirs in the broader Lango subregion, of the Lira District [28]. The AAT disease complex in cattle is generally transmitted cyclically by Glossina species tsetse flies. *Trypanosoma vivax*, on the other hand, can be spread by a wide range of haematophagous insects. A high proportion of *T. vivax* has been reported in southwestern Uganda. *Trypanosoma vivax* is more difficult to manage than the other species since it has previously been known to be mechanically transmitted by haematophagous insects, making it difficult to control even in regions with intensive vector control strategies [7]. A report of potential transmission of *T. vivax* by hematophagous insects in southeastern Uganda has been documented on the doorstep of Lira District [1]. To date, there has been no report of mechanical transmission of *T. vivax* by haematophagous insects in Lira District.

Several causes, including the gradual deterioration in state veterinary services, particularly due to adverse legislation, as well as insecurity in some parts of Uganda, may be contributing to the persistence of trypanosome infection in cattle [29]. Acaricide/insecticide and trypanocidal medicine affordability is another issue, with farmers who can afford the treatments being less likely to report cases of trypanosomiasis than those who cannot. Other likely factors include communal cattle grazing, which has been linked to a high risk of trypanosomiasis infections [17]. The continuous transmission will be achievable when cattle are grazed communally in the presence of a suitable vector because some of the cattle in the communal system will preserve the parasites. Furthermore, in addition to cattle serving as key reservoirs of trypanosomiasis, wild ungulates also play an important part in the epidemiological cycle of trypanosomiasis by serving as reservoirs. The Lango subregion that includes Lira District still contains an abundance of free-roaming wild ungulates, which are potential trypanosomiasis reservoirs, due to its closeness to the Murchison Falls Conservation Area [28].

Polymerase chain reaction amplification demonstrated that Barropok village had the highest prevalence of trypanosomiasis with 6/11 (54.5%), followed by Acanakwo A village 2/11 (18.2%) and Acungkena village 3/11 (27.3%). Microscopy as well demonstrated the highest trypanosomiasis status in Barropok. This is of high concern; therefore, measures should be put in place to control the tsetse flies that transmit this infection as well as treat the infected animals.

Noninfected animals demonstrated 21/243 (8.6%) and 222/243 (91.4%) in the abnormal and normal PCV ranges, respectively. Most of the healthy animals had normal PCV ranges. This finding is comparable with studies done elsewhere [30]. When PCV was used as a predictor value for trypanosomiasis infection and it was subjected to bivariate analysis, it gave these logistic regression values (OR = 6.041; 95% CI: 1.634-22.331; *p* < 0.05) demonstrating a statistically significant relationship between trypanosomiasis infection statuses with abnormal PCV range. The finding of this study is in agreement with other previous reports [30, 31].

## 5. Conclusion

Polymerase reaction amplification demonstrated that the prevalence of trypanosomiasis in Acanakwo A, Barropok, and Acungkena villages in Lira District, Uganda, was 11/254 (4.3%). *Trypanosoma congolense* was the most common trypanosome species with 9/11 (81.8%). Polymerase reaction amplification was the most reliable diagnostic method due to its high sensitivity and specificity as compared to the conventional microscopic method. Barropok village had the highest proportion of trypanosomiasis with 6/11 (54.5%). Polymerase reaction amplification appears to have adequate accuracy to substitute the use of a microscope where facilities allow. This study, therefore, underscores the urgent need for local surveillance schemes more especially at the grassroots in Uganda to provide data for reference guideline development needed for control of trypanosomiasis in Uganda.

## Figures and Tables

**Table 1:** ITS1 primer sequence and PCR conditions.

Primer name	Primer sequence	Trypanosomes species	Fragment size (bp)	Melting temperature
ITS1 CF	F 5′-CCGGAAGTTCACCGATATTG-3′	*T. congolense savannah*	700	Denaturation at 98°C
			700	
	R 5′-TTGCTGCGTTCTTCAACGAA-3′ [23]	*T. congolense forest*	620	Annealing at 64°C
			250	Extension at 72°C
		*T. congolense kilifi*	300	
		*T. vivax*	400	
		*T. godfreyi*		
		*T. simiae*		

**Table 2:** Comparison of PCR and PCV results.

*Trypanosoma* species	PCV ≤ 25, *n* (%) Abnormal range	PCV ≥ 26, *n* (%) Normal range	PCR, *n* (%)
*T. congolense*	3 (33.3)	6 (66.7)	9 (3.5)
*T. vivax*	0 (0.0)	1 (100.0)	1 (0.4)
*T. congolense*/*T. vivax*	1 (100.0)	0 (0.0)	1 (0.4)
Negative	21 (8.6)	222 (91.4)	243 (95.7)
Total	25 (9.8)	229 (90.2)	254 (100.0)

Footnote: *n*: number; %: percentage.

**Table 3:** Distribution of trypanosomes in the villages using microscopy.

Villages	Positive	Negative	Total
Acanakwo A	1 (1.5)	65 (98.5)	66 (26.0)
Barropok	3 (2.5)	119 (97.5)	122 (48.0)
Acungkena	1 (1.5)	65 (98.5)	66 (26.0)
Total	5 (2.0)	249 (98.0)	254 (100.0)

Footnote: *n*: number; %: percentage.

**Table 4:** Proportion of *Trypanosoma* species.

Trypanosomes species	Frequency, *N* (%)
*T. congolense*	9 (81.8)
*T. vivax*	1 (9.1)
*T. congolense*/*T. vivax*	1 (9.1)
Total	11 (100.0)

Footnote: *n*: number; %: percentage.

**Table 5:** Distribution of trypanosomes species in the villages using the PCR technique.

Villages	*T. congolense*	*T. vivax*	*T. congolense*/*T. vivax*	*Negative*	Total
Acanakwo A	2 (3.0)	0 (0.0)	0 (0.0)	64 (97.0)	66 (26.0)
Barropok	5 (4.1)	0 (0.0)	1 (0.8)	116 (95.1)	122 (48.0)
Acungkena	2 (3.0)	1 (1.5)	0 (0.0)	63 (95.5)	66 (26.0)
Total	9 (3.5)	1 (0.4)	1 (0.4)	243 (95.7)	254 (100.0)

Footnote: *n*: number; %: percentage.

**Table 6:** Relationship between PCV and trypanosomiasis infection.

Variables	Categories	Odds ratio	95% CI	*p* value
PCV	Abnormal PCV range	6.041	1.634-22.331	0.007
	Normal PCV range	1		

Footnote: CI: confidence interval; *p*: probability; OR: odds ratio; *p* ≤ 0.05 value is statistically significant under logistic regression.

## Data Availability

The tables and figures in this research article contain the data that support the study conclusions.

## References

[1] Muhanguzi D., Picozzi K., Hattendorf J. (2014). The burden and spatial distribution of bovine African trypanosomes in small holder crop-livestock production systems in Tororo District, south-eastern Uganda. *Parasites & Vectors*.

[2] Silva Pereira S., Trindade S., De Niz M., Figueiredo L. M. (2019). Tissue tropism in parasitic diseases. *Open Biology*.

[3] Dabo N. T., Maigari A. K. (2017). Soft options for effective diagnosis of African animal trypanosomiasis : a review. *International Journal of Medical Evaluation and Physical Report*.

[4] Chitanga S., Marcotty T., Namangala B., Van den Bossche P., Van Den Abbeele J., Delespaux V. (2011). High prevalence of drug resistance in animal trypanosomes without a history of drug exposure. *PLoS Neglected Tropical Diseases*.

[5] El-Sayed N. M., Hegde P., Quackenbush J., Melville S. E., Donelson J. E. (2000). The African trypanosome genome. *International Journal for Parasitology*.

[6] Holt H. R., Selby R., Mumba C., Napier G. B., Guitian J. (2016). Assessment of animal African trypanosomiasis (AAT) vulnerability in cattle-owning communities of Sub-Saharan Africa. *Parasites & Vectors*.

[7] Alingu R. A., Muhanguzi D., MacLeod E., Waiswa C., Fyfe J. (2014). Bovine trypanosome species prevalence and farmers' trypanosomiasis control methods in South-Western Uganda. *Journal of the South African Veterinary Association*.

[8] Musinguzi S. P., Suganuma K., Asada M. (2016). Full paper parasitology A PCR-based survey of animal African trypanosomosis and selected piroplasm parasites of cattle and goats in Zambia. *Journal of Veterinary Medicne*.

[9] Katiti D. (2014). *Animal African trypanosomaisis and associated cytokine profiles in naturally infected cattle in Paicho and Lakwana Subcounties, Gulu District [Ph.D. thesis]*.

[10] Široký P., Kubelová M., Modrý D. (2010). Tortoise tick Hyalomma aegyptium as long term carrier of Q fever agent Coxiella burnetii—evidence from experimental infection. *Parasitology Research*.

[11] Uilenberg G., Boyt W. P. (1998). *A Field Guide for the Diagnosis, Treatment and Prevention of African Animal Trypanosomosis*.

[12] Simwango M., Ngonyoka A., Nnko H. J. (2017). Molecular prevalence of trypanosome infections in cattle and tsetse flies in the Maasai Steppe , northern Tanzania. *Parasites & Vectors*.

[13] Alves W. P., AbrÃ D., Fern L., Facury-Filho E. J. (2017). Comparison of three methods for diagnosis of Trypanosoma (Duttonella) vivax in cattle. *Genetics and Molecular Research*.

[14] Osório A. L. A. R., Madruga C. R., Desquesnes M., Soares C. O., Ribeiro L. R. R., Costa S. C. G. D. (2008). *Trypanosoma* (Duttonella) vivax: its biology, epidemiology, pathogenesis, and introduction in the New World - a review. *Memórias do Instituto Oswaldo Cruz*.

[15] Ramírez-Iglesias J. R., Eleizalde M. C., Gómez-Piñeres E., Mendoza M. (2011). *Trypanosoma evansi*: a comparative study of four diagnostic techniques for trypanosomosis using rabbit as an experimental model. *Experimental Parasitology*.

[16] Desta M. (2014). Trypanosome infection rate of Glossina morsitans and trypanosomosis prevalence in cattle in upper Didessa valley western Ethiopia. *International Journal of Current Microbiology and Applied Sciences*.

[17] von Wissmann B., Fyfe J., Picozzi K., Hamill L., Waiswa C., Welburn S. C. (2014). Quantifying the association between bovine and human trypanosomiasis in newly affected sleeping sickness areas of Uganda. *PLoS Neglected Tropical Diseases*.

[18] Kish L. (1965). *Survey Sampling*.

[19] Greig W. A., Murray M., Murray P. K., McIntyre W. I. M. (1979). Factors affecting blood sampling for anaemia and parasitaemia in bovine trypanosomiasis. *British Veterinary Journal*.

[20] Kirchhoff L. V., Votava J. R., Ochs D. E., Moser D. R. (1996). Comparison of PCR and microscopic methods for detecting *Trypanosoma cruzi*. *Journal of Clinical Microbiology*.

[21] Njiru Z. K., Constantine C. C., Guya S. (2005). The use of ITS1 rDNA PCR in detecting pathogenic African trypanosomes. *Parasitology Research*.

[22] Kivali V., Kiyong'a A. N., Fyfe J., Toye P., Fèvre E. M., Cook E. A. (2020). Spatial distribution of trypanosomes in cattle from Western Kenya. *Frontiers in Veterinary Science*.

[23] Matovu E., Mugasa C. M., Waiswa P., Kitibwa A., Boobo A., Ndung’u J. M. (2020). Haemoparasitic infections in cattle from a *trypanosoma* brucei rhodesiense sleeping sickness endemic district of eastern Uganda. *Tropical medicine and infectious disease*.

[24] Mugasa C. M., Adams E. R., Boer K. R. (2012). Diagnostic accuracy of molecular amplification tests for human African trypanosomiasis—systematic review. *PLoS Neglected Tropical Diseases*.

[25] Radwanska M., Vereecke N., Deleeuw V., Pinto J., Magez S. (2018). Salivarian trypanosomosis: A review of parasites involved, their global distribution and their interaction with the innate and adaptive mammalian host immune system. *Frontiers in Immunology*.

[26] Truc P., Jamonneau V., N'Guessan P., N'Dri L., Diallo P. B., Cuny G. (1998). *Trypanosoma* brucei ssp. and T. congolense: mixed human infection in Côte d'Ivoire. *Transactions of the Royal Society of Tropical Medicine and Hygiene*.

[27] Nimpaye H., Njiokou F., Njine T. (2011). *Trypanosoma* vivax, T. congolense “forest type” and T. simiae: prevalence in domestic animals of sleeping sickness foci of Cameroon. *Parasite: Journal de la Société Française de Parasitologie*.

[28] Wangoola R. M., Kevin B., Acup C. A., Welburn S., Waiswa C., Bugeza J. (2019). Factors associated with persistence of African animal trypanosomiasis in Lango subregion, northern Uganda. *Tropical Animal Health and Production*.

[29] Bugeza J., Kankya C., Muleme J. (2017). Participatory evaluation of delivery of animal health care services by community animal health workers in Karamoja region of Uganda. *PloS One*.

[30] Dagnachew S., Terefe G., Abebe G. (2015). Comparative clinico-pathological observations in young Zebu (Bos indicus) cattle experimentally infected with *Trypanosoma* vivax isolates from tsetse infested and non-tsetse areas of Northwest Ethiopia. *BMC Veterinary Research*.

[31] Yayeh M., Dagnachew S., Tilahun M. (2018). Comparative experimental studies on *Trypanosoma* isolates in mice and response to diminazene aceturate and isometamidium chloride treatment. *Heliyon*.

